# No Difference in Outcomes After Arthroscopic Bankart Repair With Remplissage or Arthroscopic Latarjet Procedure for Anterior Shoulder Instability

**DOI:** 10.1016/j.asmr.2021.12.011

**Published:** 2022-04-18

**Authors:** Eoghan T. Hurley, Christopher A. Colasanti, Nathan A. Lorentz, Bogdan A. Matache, Kirk A. Campbell, Laith M. Jazrawi, Robert J. Meislin

**Affiliations:** New York University Langone Health, New York, New York, U.S.A.

## Abstract

**Purpose:**

To evaluate the outcomes of arthroscopic Bankart repair with remplissage (ABRR) compared with the arthroscopic Latarjet (AL) procedure for anterior shoulder instability in patients with a labral tear and a concomitant engaging Hill-Sachs lesion.

**Methods:**

A retrospective review of patients who underwent either ABRR or the AL procedure for a diagnosis of anterior shoulder instability with a concomitant engaging Hill-Sachs lesion between 2011 and 2019 was performed. Recurrent instability, the visual analog scale score, the Subjective Shoulder Value, the Western Ontario Shoulder Instability score, patient satisfaction, willingness to undergo surgery again, and return to work or sport were evaluated.

**Results:**

Our study included 41 patients treated with ABRR and 26 treated with the AL procedure. At final follow-up, there was no difference between patients who underwent ABRR and those who underwent the AL procedure in the reported Western Ontario Shoulder Instability score (21.8% vs 28.2%, *P* = .33) or any of its components, the visual analog scale score (0.9 vs 1.4, *P* = .32), the Subjective Shoulder Value (78.4 vs 74.5, *P* = .6062), the rate of satisfaction (81.6% vs 85.6%, *P* = .54), or whether patients would undergo surgery again (81.6% vs 96.1%, *P* = .16). Overall, 5 patients in the ABRR group and 2 patients in the AL group had recurrent instability events (12.2% vs 7.8%, *P* = .70), with no significant difference in the rate of recurrent dislocation (12.2% vs 3.8%, *P* = .39).

**Conclusions:**

In patients with anterior shoulder instability and a concomitant Hill-Sachs lesion, both ABRR and the AL procedure were shown to be reliable treatments, with a low rate of recurrent instability and excellent patient-reported outcomes in appropriately selected patients. However, our study could not determine whether there was critical glenoid bone loss in patients undergoing ABRR, and surgeons should still exercise caution in performing ABRR in patients with high-grade glenoid bone loss or in those with failed prior stabilizations.

**Level of Evidence:**

Level III, retrospective cohort study.

Anterior shoulder instability is a common clinical problem, affecting up to 2% of the general population.^1^^,^^2^ Arthroscopic Bankart repair (ABR) is the most widely performed shoulder stabilization procedure both in the United States and globally.^3^ However, in the presence of an engaging Hill-Sachs lesion, ABR in isolation has been shown to be associated with a greater than 30% failure rate at 10-year follow-up.^4^, ^5^, ^6^ Options in this setting include the Latarjet procedure and arthroscopic Bankart repair with remplissage (ABRR).^7^

The Latarjet procedure is indicated in patients with anterior shoulder instability who have a high risk of failure of ABR, including those with multiple previous dislocations, an engaging Hill-Sachs lesion, and glenoid bone loss.^8^ Historically, the Latarjet procedure has been performed via an open approach, but an arthroscopic technique, described by Lafosse et al.,^9^ has recently gained popularity owing to its minimally invasive approach and improved intra-articular visualization. These factors potentially allow for more accurate graft placement, less postoperative stiffness, fewer wound complications, and a quicker rehabilitation.^9^, ^10^, ^11^ However, there is a concern that this technically challenging procedure may result in a higher complication rate among its adopters.^10^^,^^11^

ABRR is an alternative option to the Latarjet procedure for patients with anterior shoulder instability and an engaging Hill-Sachs lesion.^12^, ^13^, ^14^ Originally described by Purchase et al.,^15^ ABRR involves tenodesis of the infraspinatus tendon and posterior capsule into the humeral defect, thus rendering it extra-articular and preventing engagement. The primary concern with performing this procedure is the risk of reduced range of motion of the shoulder postoperatively.

There is scant literature comparing these procedures, with a recent meta-analysis by Hurley et al.^16^ finding no difference between the open Latarjet procedure and ABRR; however, they did not include any studies evaluating the arthroscopic Latarjet (AL) procedure. The purpose of this study was to evaluate the outcomes of ABRR compared with the AL procedure for anterior shoulder instability in patients with a labral tear and a concomitant engaging Hill-Sachs lesion. Our hypothesis was that there would be no significant difference in recurrence rates or functional outcomes between the 2 procedures.

## Methods

### Patient Selection

This study received ethical approval from our institutional review board. A retrospective review was carried out to identify all patients who underwent ABRR or the AL procedure between 2011 and 2019. The operative notes of all patients who underwent ABRR or the AL procedure for shoulder instability were analyzed. Patients made an informed decision regarding their treatment preference, those with high-grade bone loss or failed prior stabilization were cautioned of the potentially higher recurrence rate with ABRR, and the risk of complications after the AL procedure was discussed. All patients underwent preoperative magnetic resonance imaging at our institution evaluated by a musculoskeletal radiologist. We included all patients who underwent either ABRR or the AL procedure, were aged older than 16 years at the time of surgery, were skeletally mature, and had a minimum follow-up period of 24 months postoperatively.

### Surgical Technique

##### ABR With Remplissage

ABRR was performed with the patient under general anesthesia and an interscalene nerve block in the lateral decubitus position by 3 sports medicine–trained attendings (K.A.C., L.M.J., and R.J.M.). In some cases, a lateral distractor (Lateral Jack; Smith & Nephew, Andover, MA) was used to improve intra-articular visualization. A standard posterior portal was created first and was used to perform a diagnostic arthroscopy. Glenoid bone loss was calculated in all patients by the operating surgeon at the time of arthroscopy.^17^ The surgical technique was performed as described by Purchase et al.^15^ The Hill-Sachs lesion was identified, and an accessory 7-o’clock portal was created for preparation of the Hill-Sachs bed. After insertion of an 8.25-mm cannula (Twist-In; Arthrex, Naples, FL) into each of the posterior portals, two 4.75-mm bio-composite anchors doubly loaded with suture tape (Corkscrew; Arthrex) were inserted into the defect. The cannulas were then backed out of the posterior capsule, and a bird beak–type instrument was used to pierce the capsule and retrieve the corresponding suture limbs in a horizontal mattress–type configuration. After completion of the ABR with a minimum of 3 anchors, the remplissage sutures were tied and cut in a blinded fashion in the subacromial space.

##### AL Procedure

The AL procedure was performed with the patient under general anesthesia and an interscalene nerve block in the beach-chair position with the addition of an arm positioner (Spider2; Smith & Nephew). The surgical technique followed the steps outlined by Lafosse et al.^9^ and modified by the senior surgeon (R.J.M.). A standard posterior portal was created first and was used to perform a diagnostic arthroscopy. Glenoid bone loss was calculated in all patients by the operating surgeon at the time of arthroscopy.^17^ The surgical technique was performed as described by Lafosse et al. Six portals were used for this technique, which in summary consisted of the following surgical steps: (1) preparation of the anterior glenoid neck; (2) rotator interval release; (3) anterior, superior, and posterior subscapularis release; (4) exposure of the coracoid and conjoint tendon; (5) subdeltoid bursoscopic coracoacromial ligament and pectoralis minor release; (6) coracoid osteotomy and graft preparation; (7) subscapularis split; and (8) graft fixation using two 3.5-mm partially threaded, cannulated cancellous screws. Minor changes were developed by the senior author over the years based on clinical experience with this procedure to improve its safety profile and time efficiency. These changes include performing decortication of the coracoid base with an arthroscopic burr prior to osteotomy to reduce the risk of fracture propagation.

### Rehabilitation Protocol

The rehabilitation protocol was the same for all patients. Postoperatively, the shoulder was placed in a sling for 3 weeks after the AL procedure and for 6 weeks after ABRR while allowing non-resisted activities of daily living without excessive elevation or external rotation of the shoulder. Patients immediately began physiotherapy, which continually increased in intensity over the next 9 weeks. A return to resistance training was allowed after 12 weeks, whereas a return to full contact and competition would usually follow within the next 3 months. Athletes were cleared to return to play (RTP) when they achieved appropriate thresholds for time, strength, range of motion, and pain reduction.

### Data Collection and Clinical Outcomes

Data on patient characteristics and preoperative demographic characteristics were collected, including age, sex, laterality, glenoid bone loss, Hill-Sachs defect, and previous shoulder operations. Intraoperative and postoperative complications were recorded. Evaluation of postoperative patient-reported outcomes was carried out through a telephone survey including the visual analog scale (VAS) score, the Subjective Shoulder Value, the Western Ontario Shoulder Instability index score, satisfaction, and whether patients would undergo the same surgical procedure again. This was performed by unblinded 2 authors (E.T.H. and C.A.C.), both of whom are orthopaedic residents. Additionally, the rate and timing of RTP, rate of return to work (RTW), and Shoulder Instability–Return to Sport After Injury (SIRSI) score were evaluated. Finally, recurrent instability (including dislocations and subluxations) was recorded.

### Statistical Analysis

All statistical analysis was performed using GraphPad Prism (version 8.3; GraphPad, La Jolla, CA). For all continuous and categorical variables, descriptive statistics were calculated. Continuous variables were reported as weighted means and estimated standard deviations, whereas categorical variables were reported as frequencies with percentages. Categorical variables were analyzed using the Fisher exact or χ^2^ test. The independent or paired *t* test was performed for normally distributed variables, and the nonparametric Mann-Whitney *U* test or Wilcoxon signed rank test was performed to compare continuous variables. *P* < .05 was considered statistically significant.

## Results

### Patient Demographic Characteristics

Overall, 54 patients were treated with ABRR and 36 patients were treated with the AL procedure; of these patients, 41 and 26, respectively, were available for follow-up. There were no significant differences in demographic variables between the groups, except for the amount glenoid bone loss and rate of prior surgery, which were both higher in the AL group. A comparison of patient demographic characteristics between the ABRR and AL groups is further illustrated in Table 1.

**Table 1:** Patient Characteristics

	ABRR	AL Procedure	*P* Value
n	41	26	—
Age, yr	29.6 ± 10.8 (16-59)	32.3 ± 12.7 (18-60)	.3553
Male sex	28 (68.3)	21 (80.8)	.2616
Glenoid bone loss, %	7.3 ± 7.8 (0-20)	19.1 ± 4.7 (10-30)	<.0001
Engaging Hill-Sachs lesion	41 (100)	26 (100)	>.99
Prior surgery	2 (5.0)	11 (42.3)	.0002
Follow-up, mo	58.5 ± 24 (24-105)	52 ± 23.4 (24-90)	.2794

NOTE. Data are presented as number (percentage) or mean ± standard deviation (range).

ABRR, arthroscopic Bankart repair with remplissage; AL, arthroscopic Latarjet.

### Functional Outcomes

At final follow-up, there was no difference between patients who underwent ABRR and those who underwent the AL procedure in the reported Western Ontario Shoulder Instability score (21.8% vs 28.2%, *P* = .33) or any of its components, the VAS score (0.9 vs 1.4, *P* = .32), the VAS score during sports (1.7 vs 2.4, *P* = .29), the Subjective Shoulder Value (78.4 vs 74.5, *P* = .32), the SIRSI score (69.3 vs 62.8, *P* = .34), the rate of satisfaction (81.6% vs 85.6%, *P* = .54), or whether patients would undergo surgery again (85.4% vs 96.1%, *P* = .16). A comparison of patient-reported outcomes between the groups is shown in Table 2.

**Table 2:** Functional Outcomes

	ABRR	AL Procedure	*P* Value
WOSI score, %	21.8 ± 25.2 (14.1-29.5)	28.2 ± 27.1 (19.9-36.5)	.3288
WOSI component score, %			
Physical	22 ± 25 (14.3-29.7)	28.7 ± 26.3 (20.6-36.8)	.2987
Sport	18.4 ± 23.1 (11.3-25.5)	25.9 ± 28.7 (17.1-34.7)	.2432
Lifestyle	17.1 ± 25.3 (9.3-24.9)	22.1 ± 25.9 (14.1-30.1)	.4376
Emotional	26.8 ± 29.3 (17.8-35.8)	32.6 ± 31.7 (22.8-42.4)	.3553
VAS score	0.9 ± 1.9 (0.3-1.5)	1.4± 2.1 (0.8-2.0)	.3174
VAS score for sport	1.7 ± 2.5 (0.9-2.5)	2.4 ± 2.8 (1.5-3.3)	.2904
SSV	78.4 ± 19.4 (72.6-84.2)	74.5 ± 23.2 (67.5-81.5)	.4603
SIRSI score	69.3 ± 24.7 (61.5-77.1)	62.8 ± 29.9 (53.5-72.1)	.3373
Satisfaction, %	81.6 ± 30 (72.4-90.8)	85.6 ± 17.7 (80.2-91)	.5411
Would repeat surgery	35 (85.4)	25 (96.1)	.1595

NOTE. Data are presented as number (percentage) or mean ± standard deviation (95% confidence interval).

ABRR, arthroscopic Bankart repair with remplissage; AL, arthroscopic Latarjet; SIRSI, Shoulder Instability–Return to Sport After Injury; SSV, Subjective Shoulder Value; VAS, visual analog scale; WOSI, Western Ontario Shoulder Instability.

### RTP and RTW rates

Overall, there was no significant difference in the total rate of RTP (60.9% vs 66.7%, *P* = .70) or the timing of RTP (7.7 months vs 7 months, *P* = .17). Additionally, there was no significant difference in the total rate of RTW (100% vs 100%, *P* > .99). A comparison of RTP and RTW rates between the groups is shown in Table 3.

**Table 3:** Rates of RTP and RTW

	ABRR	AL Procedure	*P* Value
RTP	14 of 23 (60.9)	12 of 18 (66.7)	.7021
RTP timing, mo	9.1 ± 5.2 (8.2-10)	6.7 ± 2.8 (5.1-8.3)	.1660
RTW	29 of 29 (100)	17 of 17 (100)	>.99

NOTE. Data are presented as number (percentage) or mean ± standard deviation (95% confidence interval).

ABRR, arthroscopic Bankart repair with remplissage; AL, arthroscopic Latarjet; RTP, return to play; RTW, return to work.

### Recurrent Instability

Overall, 5 patients in the ABRR group and 2 patients in the AL group had recurrent instability events (12.2% vs 7.7%, *P* = .70), with no significant difference in the rate of recurrent dislocation between the groups (12.2% vs 3.8%, *P* = .39). Further analysis of the ABRR group revealed no significant difference in recurrence rates between patients with greater than 10% glenoid bone loss and those with less than 10% glenoid bone loss (14.3% [3 of 21] vs 9.5% [2 of 20], *P* > .99) or between those with greater than 15% glenoid bone loss and those with less than 15% glenoid bone loss (18.2% [2 of 11] vs 10% [3 of 30], *P* = .60). Additionally, there was no difference in the recurrence rate between patients who underwent ABRR and those who underwent the AL procedure for those with greater than 10% glenoid bone loss (14.3% vs 7.7%, *P* = .66) or those with greater than 15% glenoid bone loss (18.2% vs 8%, *P* = .57). A comparison of recurrent instability between the ABRR and AL groups is presented in Table 4.

**Table 4:** Recurrent Instability Rates

	ABRR	AL Procedure	*P* Value
Total recurrence	5 (12.2)	2 (7.7)	.6972
Redislocation	5 (12.2)	1 (3.8)	.3925
Subluxation	0	1 (3.8)	.4098

NOTE. Data are presented as number (percentage).

ABRR, arthroscopic Bankart repair with remplissage; AL, arthroscopic Latarjet.

### Complications

There was no significant difference in the overall complication rate between ABRR and the AL procedure (4.9% vs 7.7%, *P* = .57). In the ABRR group, 1 patient had a prominent suture anchor at the glenoid causing pain, which was loose and had to be removed at 2 months postoperatively. In the AL group, 1 patient (3.3%) required revision to treatment with distal tibial allograft for graft fracture and dislocation at 2 months postoperatively and another patient (3.3%) had drainage from one of the portals and was treated with antibiotics for a suspected infection. None of the postoperative radiographs showed malpositioning in either group.

## Discussion

The most important finding of our study was that in patients with a concomitant engaging Hill-Sachs lesion, both ABRR and the AL procedure are reliable comparable options, with low rates of recurrent instability and excellent patient-reported outcomes. However, there were differences in the amount of glenoid loss between the 2 cohorts, and thus, the similar outcomes are with appropriate indication for either procedure. Further research is still needed to define what the amount of critical glenoid bone loss is in patient undergoing ABRR.

ABRR and the AL procedure are both recent advancements in the arthroscopic management of anterior shoulder instability, with Purchase et al.^15^ describing ABRR in 2008 and Lafosse et al.^9^ describing the AL procedure in 2007. ABRR was developed to address the engaging Hill-Sachs lesion, which has increasingly been recognized as a risk factor for postoperative recurrence. ABRR fills the Hill-Sachs defect using the infraspinatus and posterior-inferior capsule. As a result, engagement of the Hill-Sachs lesion is prevented and the lesion remains “on track,” which is not addressed by an ABR alone. This concept has been supported by biomechanical and clinical data: Lazarides et al.^13^ performed a systematic review of biomechanical data and found that ABRR consistently prevented engagement of the Hill-Sachs lesion on the anterior glenoid in most of the studies in the literature. Thereafter, Hurley et al.^16^ found that ABRR reduced the recurrence rate in the setting of a Hill-Sachs lesion by approximately 80% compared with ABR alone.

In contrast to ABRR, the AL procedure widens the glenoid articular surface while simultaneously providing stability by way of the sling effect provided by the transposed conjoint tendon; both of these factors reduce the chances of the Hill-Sachs lesion engaging.^18^^,^^19^ The arthroscopic approach has been advocated owing to its minimally invasive approach, which potentially results in decreased stiffness, decreased wound complications, and a quicker rehabilitation.^9^, ^10^, ^11^ However, there is a concern that this technically challenging technique may result in a higher complication rate especially during the early learning phase.^10^^,^^11^ Hurley et al.^11^ found in their meta-analysis that both the open approach and the arthroscopic approach result in similar clinical outcomes, with similar recurrence rates, but lower pain scores in patients treated with the AL procedure. Furthermore, there was no difference in complication rates between the 2 procedures. However, Hurley et al. did note the technical complexity of the arthroscopic approach and indicated that it may be advisable for this approach to be performed only in high-volume centers by experienced arthroscopists.

Overall, there was no significant difference in any functional outcome measure between patients undergoing ABRR and those undergoing the AL procedure, with a high rate of satisfaction and willingness to undergo the procedure again if required. Low pain scores were reported in both groups, which may be a result of the minimally invasive arthroscopic approach, which in particular has been a reported benefit of performing the Latarjet procedure arthroscopically.^11^ Furthermore, no differences in return to premorbid function were observed, with almost all patients able to RTW. However, the rate of RTP was slightly lower, although this may be because of the slightly older age group in this study, given that both procedures have been shown to result in high rates of RTP among young athletes.^20^, ^21^, ^22^ The SIRSI score, which assesses athletes’ psychological confidence in the shoulder, was also similar between the 2 groups.

Overall, despite more severe glenoid bone loss and more patients having undergone prior surgical procedures, the AL procedure showed a slightly lower rate of recurrent instability; however, this was not statistically significant. The amount of glenoid bone loss in the setting of an “off-track” Hill-Sachs lesion that is critical to failure after ABRR is still undefined. Yang et al.^23^ found in their series that among patients who had greater than 10% glenoid bone loss, the outcomes were worse in those who received ABRR than in those who received the Latarjet procedure. In contrast, in our series, half of the patients had greater than 10% glenoid bone loss and there was no difference in the recurrence rate compared with patients with less than 10% glenoid bone loss. The Latarjet procedure has been shown to be a viable procedure for patients with a failed prior stabilization, with previous studies showing similar results to those in patients with primary instability.^24^^,^^25^ Although it is unclear how a prior failed ABR affects the outcomes of ABRR, further soft-tissue damage may predispose these patients to a higher risk of failure.^26^ Both procedures appear to result in similar outcomes with their appropriate indications, but surgeons should be cautioned about choosing ABRR in patients with severe glenoid bone loss until this is better defined.

There were 1 complication in the group of patients undergoing ABRR in our series, highlighting that it is a safe procedure, and on the basis of the literature, it does not appear to confer any additional risk over ABR alone. There were 2 complications in the group undergoing the AL procedure in our series, one of which required a revision within 90 days because of a fractured coracoid graft and was treated with distal tibial allograft. The other patient was treated for a suspected wound infection, which resolved with antibiotics. There is a concern regarding the Latarjet procedure and its associated complications and morbidity, with a high rate reported in the literature: Griesser et al.^27^ found a complication rate of 30% after the Latarjet procedure. Thus, careful counseling of patients is warranted owing to these complications.

### Limitations

There were several potential limitations and sources of bias in this study. First, this study was a retrospective analysis with a follow-up rate of approximately 75% and was thus subject to potential bias. Additionally, there were differences in the amount of glenoid loss between the 2 cohorts, although this finding represents differences in clinical indications. The intraoperative measurement of glenoid bone loss is a limitation because it prevents the ability to assess inter-rater reliability and is not as accurate as measurement by preoperative imaging; moreover, because multiple surgeons performed these measurements, some variability may have been introduced. Furthermore, although postoperative loss of range of motion may be a concern with both procedures, this was not assessed in our study. We did not evaluate the actual glenoid track itself because several patients underwent surgery prior to establishment of the new definition. Finally, a post hoc power analysis was performed revealing that 1,386 patients would be required to detect a difference in the rate of recurrent dislocation, with an α of .05 and a power of 0.8. A post hoc power analysis revealed that 352 patients would be required to detect a difference in patients with greater than 15% glenoid bone loss.

## Conclusions

In patients with anterior shoulder instability and a concomitant Hill-Sachs lesion, both ABRR and the AL procedure were shown to be reliable treatments, with a low rate of recurrent instability and excellent patient-reported outcomes in appropriately selected patients. However, our study could not determine whether there was critical glenoid bone loss in patients undergoing ABRR, and surgeons should still exercise caution in performing ABRR in patients with high-grade glenoid bone loss or in those with failed prior stabilizations.

## Supplementary Data

ICMJE author disclosure forms
